# Spinal deformity

**DOI:** 10.4103/0019-5413.61725

**Published:** 2010

**Authors:** Myung-Sang Moon, Bong-Jin Lee, Sung-Soo Kim

**Affiliations:** 1 Department of Orthopaedic Surgery, Cheju Halla General Hospital, Jeju, Korea; 2 Department of Orthopaedic Surgery, Catholic University of Korea, Seoul, Korea

The vertebral column is an aggregate of articulated, superimposed segments, each of which is a functional unit. The function of the vertebral column is to support a man in upright position, mechanically balance to conform to the stress of gravity, permit locomotion and assist in purposeful movements.

The head is located over the body of the sacrum, and the spine in an upright manner bears an axial load to support the body. Loss of this spinal balance produces a position that is at a biomechanical disadvantage. To stand upright and look forward while standing and walking, the patient with sagittal plane imbalance causes back muscle to strain to successfully or unsuccessfully reduce a patient's sideways tilt. The additional energy expenditure associated with standing and walking in patient with a spinal deformity leads to reduced functional capacity including pulmonary function and a poorer quality of life.

The definition and scope of spinal deformity continues to evolve. Certainly, the term spinal deformity includes conditions such as idiopathic adolescent scoliosis, congenital scoliosis, post-traumatic deformities and other adult spinal deformity including post-infective kyphosis. In our region, one of the most common severe deformities is the post-infective kyphosis particularly in the less privileged countries.

In ancient times, Hippocrates and his successors treated scoliosis by traction and counter-traction on his bench, and Ambroise Paré, in the 16^th^ century, was credited with describing the first use of bracing to treat scoliosis. The basic methods of treatment throughout the ages have consisted of traction, support and more or less vigorous redressent plus exercise and massage. In 1911, Hibbs^1^ and Albee^2^ introduced their spinal fusion for tuberculosis of spine, and then fusion method was adopted for scoliosis management. However, there was no steady progress in the management of spinal deformity until 1945 when Smith-Petersen^3^ introduced a spinal osteotomy procedure which was modified by the several surgeons. One disadvantage for surgeons at the time was that internal fixation device for the osteotomy was not available. In 1955, Harrington introduced the hooks and rods system which revolutionalized the deformity correction surgery. Halo distraction apparatus, developed by Nickel and Perry in 1959 could be utilized for a severely deformed and rigid spine.^4^

Thereafter, Luque's segmental fixation system for scoliosis^5^,^6^ and Roy-Camille's pedicle screw and plate system for fracture fixation were introduced.^7^ The improved Roy-Camille's system later developed into Cotrel and Dubousset (C-D) system (1984).^8^,^9^ Spinal deformity correction surgery, together with the development of new fixation devices, has seen remarkable technical evolution since early 1980. The indications for spinal osteotomy have been broadened for last 20 years to include patients with congenital kyphosis and scoliosis, adult scoliosis, and post-traumatic and post-infective kyphosis. More effective instrument-aided deformity correction and stabilization after osteotomy or spondylectomy could be possible by utilizing the Hartshill segmental stabilization system,^5^ hook and/or pedicle screw system since early 1990. Since early 2000 the mono- and bi-plane spinal deformities could be successfully corrected by combined two stage anterior release and posterior osteotomy procedure or one-stage posterior spondylectomy.

Although the surgical deformity correction is well documented, controversy has arisen as to the true outcome of such an expensive, technically demanding, complication fraught procedure. Some would argue that the procedure is primarily a cosmetic one that mainly allows patients to look better by standing. In summary, most spine surgeons would argue that the procedure restores the sagittal balance as well as possibly correcting decompensated coronal balance that ultimately reduces the energy required by the patient to stand and walk.

A high level of patient satisfaction can be achieved if performed by experienced technically skilled surgeons, and a significant risk is understood to exist by the patient. The current authors’ belief is that in the recent years the corrected spine not only improves the spinal column function but also the cosmesis and quality of life.

Post-infective spinal deformity is a preventive disease. However, if the disease is neglected it results in esthetically unacceptable deformity and needs surgical care. Non-paralytic young patients tend to have very high esthetic demands, and to drive decision making rather than pure surgical indications. This has led to the development of safe and effective corrective surgical procedures for unsightly kyphosis, but each patient must be considered individually.

Healed tuberculous kyphosis in an adult is very rigid and angled acutely. The spinal cord has poor tolerance to the traction. The objectives of the corrective surgery for severe rigid tuberculous kyphosis are: to meet the patient's esthetic demands by the surgical realignment of the spine from severe to normal without impairing neurologic function, to maintain the cord function by preventing late onset paralysis, to improve pulmonary function, and to promote social rehabilitation through better outlook.

The important precautions with surgical management are:

1. Careful preoperative patient's evaluation
2. Delicate and careful cord exposure
3. Greatest care and delicacy should be paid in insertion of hooks, wires, and screws and the intrusion of screws should be minimal if possible
4. In case of anterior surgery, the vessel ligation should be done only on one side and always on convexity
5. Excessive distraction should never be done because inadvertant stretch and kinking can easily damage cord circulation
6. Cord monitoring and/or wake-up test should be done during surgery
7. Hypotension should be avoided during surgery to maintain the normal blood flow to the cord

Severe post infective deformity is not only a clinical problem but also a cosmetic problem; with increased deformity, pain appears and neurologic deficit may develop or increase if untreated. Surgical restoration of anatomic alignment reduces the rate of instrumentation failure, and increases the fusion rates.

O'Brien *et al*.^6^ (1971) and Yau *et al*.^10^ (1973) reported that a halo-pelvic traction as the safest and efficient method for the correction of the rigid kyphosis and scoliosis. However, later they concluded that their corrective procedure had a small reward for such a major undertaking, and the hazard of deformity correction outweighs the gain. Hence it should not be carried out for cosmetic gains.

A two-stage correction operation-anterior release and decompression and posterior correction has been commonly used for angular kyphosis and kyphoscoliosis of the thoracic and thoracolumbar spine. The single posterior approach has been used rarely until end of 1990. However, since Kawahara *et al*.^11^ and Shimode *et al*.^12^ reported that they successfully performed corrective en bloc spondylectomy for the severe kyphotic deformity, the procedure drew attention. Although the surgical performance was known to be technically laborious it offered good correction without jeopardizing the integrity of the spinal cord. The current authors, however, recommend only the decompression surgery for Pott's paraplegics with severe kyphosis, and not the total en bloc spondylectomy procedure.^13^,^14^

For spinal stabilization after deformity correction in the past few decades, pedicle screw placement has brought in a genuine scientific revolution in the surgical care of the spinal disorders. There is still concern that thoracic pedicle screws carry more risk than wires or hooks do, but to date no reports have suggested the thoracic screw technique is associated with a higher rate of neurologic deficit.

Another complication unique to pedicle screw is the risk to the great vessel. The percentages of misplaced screws inserted under fluoroscopy were obtained and compared to the percentage of misplaced screws inserted under computer assisted image guidance reported in the literature. The result was that there was no significant difference between two techniques. The computer assisted image guidance system demonstrated the improved accuracy with the placement of screw. However, the learning curve was fairly steep, and major pedicle violations were initially 12.5% and then improved 7.5%.^15^ It is the authors’ review that robot and/ or computer-based technology will result in more accurate and safe pedicle screw placement.

## CORRECT PEDICLE PLACEMENT, SPINAL OSTEOTOMY AND DEFORMITY CORRECTION

Debates exist regarding the optimum implant method of fixation. The use of thoracic pedicle screws results in potentially more correction than can be achieved with hooks and wires or with a hybrid construct of hooks and screws. It is debatable whether a 55 ~ 65% correction is of any clinical importance, but if the pedicle screw technique can allow the surgeon to avoid an anterior operation or save distal fusion segments, there is a substantial benefit. Also, the use of pedicle screw implants facilitates the treatment of severe deformity, defined as a scoliosis curve of >100° or sagittal kyphosis of >120°. In addition, halo-gravity and halo-femoral traction may have role, and vertebral column resection is an option for these severe deformities.

Although anterior release has been considered a necessary and helpful ingredient in the correction of large curves, there is currently a strong trend away from it and towards more reliance on posterior release (osteotomy and spinal shortening) techniques.

Those wishing to use the pedicle screw fixation must have adequate training, and if image guidance is used, it should be relegated to an adjunctive role than the primary means of determining an entry site and trajectory.

## CORRECTIVE SURGERY AND NEUROLOGICAL COMPLICATION

The reported incidence of neurologic deficits following pedicle subtraction or V-shaped osteotomy, and spondylectomy are known to be around 12% (range 0~15.2%).^7^ Congenital scoliosis often associates with the small spinal cord that can increase the risk for neurological complication following osteotomy or spondylectomy. Thus, this information should be included in the treatment strategy.

## SPINAL OSTEOTOMY AND DURAL STRETCH OR BUCKLING

Lehmer *et al*.^9^ recommended in their report on posterior transvertebral osteotomy that correction at any one level should not exceed approximately 35°. Otherwise dural buckling, which may require dural plasty may occur.

## SPINAL COLUMN SHORTENING AND CORD FUNCTION

The ideal size of the longitudinal spondylectomy, en bloc hemi- or total spondylectomy in the correction of the spinal deformity in relation with the cord function has rarely been discussed, and there is no consensus view. In the normal spine, the cord length, spinal canal and anterior spinal column length are equal. Kawahara *et al*.^11^ concluded that 20% column shortening in spinal tumor surgery might be safe, while Kobayashi *et al*, concluded that the safety limit for column shortening in dog was 12.5 mm (62.5%).^16^

## LIGATION OF RADICULAR ARTERY AND CORRECTIVE SPINE SURGERY

The ligation of the radicular artery is the key procedure in carrying a safe and bloodless surgery, but can also affect the cord circulation.^11^,^17^ Therefore, Luque stressed the importance of leaving the segmental vessels intact for the blood supply of the spinal cord in the posterior decancellation technique for multiple vertebrectomy.^5^ However, Kawahara and Tomita^9^,^11^ reported no neurologic complication in a series of total enbloc spondylectomy involving one to three segments after bilateral ligation of the radicular artery. No neurologic complication occurred in Kawahara *et al*'s series.^11^ Toribatake found, in a cat model, that ligation of the Adamkiewicz artery reduced spinal cord blood flow by approximately 81% of the control value, but such decreased blood did not influence spinal cord-evoked potential.^17^ The spinal cord completely compensates for the ligation of one or two radicular arteries because of the abundant arterial network around both dura mater and the spinal cord.

## ADULT SPINAL DEFORMITY

Surgical treatment of spinal deformity demands a solid fusion, and a long construct from the thoracic spine to the sacrum is often needed. The results supported sagittal plane balance (not coronal plane correction/ balance) as the primary radiographic factor in determining the outcome. The current authors’ view is that restoration of sagittal alignment together with coronal alignment is essential to minimize and/or delay the development of adjacent segmental disease.

Complications of adult spinal deformity surgery have been the focus of many presentations with additional data on catastrophic failure of the (1) proximal adjacent segment in pedicle screw construct, (2) women over the age of 60 years with sagittal imbalance, (3) obesity, (4) osteoporosis, and (5) substantial sagittal plane correction.

## INSTRUMENT-AIDED DEFORMITY CORRECTION IN CHILDREN

Since the introduction of Harrington rods traditional surgical correction of spinal deformity has involved relatively long instrumentation and fusion techniques, producing a straighter but stiff spine. In the infant, this approach leads to a shorter trunk. Current surgical techniques may also have an adverse effect on pulmonary function. Non-fusion technique in the growing spine, to maximize or modulate future growth potential, is being explored. Their potential advantages include obviation of the need for early fusion and countering of the resultant relative axial shortening from the spinal arthrodesis.

## IS PROPHYLAXIS OF DEFORMITY PROGRESSION POSSIBLE?

Congenital, natural attritional and/or disease-related spinal changes all may lead to cosmetically unacceptable deformity together with disability. Deformity correction surgery aims, primarily, not only to improve the spinal function but also to improve spinal cosmesis. However, it is generally believed by laymen that the main health care gain in the correction of idiopathic scoliosis and other acquired spinal deformities is cosmetic.

Postoperative assessment goal is to correlate the patient's outcomes with postoperative restoration of sagittal balance and complications. Residual coronal or sagittal imbalances were significantly associated with poorer patient satisfaction.

Computer aided or robot spine surgery is a hot and complex subject. The currently available image-guided surgery system has proved valuable in conducting safe pedicle screw placement. For this it is strongly recommended that all spine surgeons have a basic training on how to carry out the robot and/or computer guided surgeries in the coming years.

For early detection and care of the spinal deformities, national and/or community-based surveys by the school nurses and/or community healthcare personnel is recommended.

Successful surgeries can be attributed to the development of new upgraded surgical skill, surgical implants, robot and/ or computer assistance and cord monitoring.

The symposium on this issue has 2 review articles on kyphosis in spinal tuberculosis by Jain AK and congenital scoliosis by Debonath U. An article on pedical morphometry in patients of adolescent idiopathic scoliosis (AIS) by upendra B discusses the variations in the size of pedicles on cancave and canvex side and by Rajasekaran, *et al*., the advantages of ISO-3CD navigation in placement of pedicle screw in thoracic and cervical spine. Canavese *et al.* discusses the use of vacuum assisted closure in post operative infection following instrumented correction of spinal deformity in children.

Through sowing we can harvest good crops. Let us lay down the fertile academic soil through exchange of thoughts. Continuous efforts should be made to upgrade the surgical technique.

## References

[1] Hibbs RA (1911). An operation for Pott's disease of the spine. J Am Med Assoc.

[2] Albee FH (1911). Transplantation of a portion of the tibia into spine for Pott's disease. J Am Med Assoc.

[3] Smith-Petersen MN, Larson CB, Aufranc OE (1945). Osteotomy of the spine for correction of flexion deformity in rheumatoid arthritis. J Bone Joint Surg.

[4] Perry J, Nickel VL (1959). Total cervical spine fusion for neck paralysis. J Bone Joint Surg Am.

[5] Luque ER, Luque ER (1984). Editor's commentary Segmental spinal instrumentations Thorofare NJ Slack.

[6] O'Brien JP, Hodgson AR, Smith TK, Yau ACMC (1971). Halo-pelvic traction. A preliminary report of external skeletal fixation for correcting deformities and maintaining fixation of the spine. J Bone Joint Surg Br.

[7] Roy-Camille R, Berteaux D, Saillant J (1977). Unstable fractures of the spine. IV. Stablization methods and their results. B. Surgical methods. 1. Synthesis of the injured dorso-lumbar spine by plates screwed into vertebral pedicles. Rev Chir Orthop Reparatrice Appar Mot.

[8] Moon MS (2007). Tuberculosis of the spine-contemporary thoughts on current issues and perspective views. Curr Orthop Relat Res.

[9] Lehmer SM, Kappler L, Buscup RS, Enker P, Miller SD, Steffee AD (1994). Posterior transvertebral osteotomy for adult thoracolumbar kyphosis. Spine.

[10] Yau AMC, Hsu LCS, O'Brien JP, Hodgson AR (1974). Tuberculous kyphosis; correction with spinal osteotomy, halopelvic distraction, and anterior and posterior fusion. J Bone Joint Surg (Am).

[11] Kawahara N, Tomita K, Baba H, Kobayashi T, Fujita T, Murakami H (2001). Closing-opening wedge osteotomy to correct angular kyphosis deformity by a single posterior approach. Spine.

[12] Shimode M, Kojima T, Sowa K (2002). Spinal wedge osteotomy by a single posterior approach for correction of severe and rigid kyphosis or kyphoscoliosis. Spine.

[13] Kim KD, Patrick Johnson J, Bloch BS O, Masciopinto JE (2001). Computer-assisted pedicle screw placement: An intro feasibility study. Spine.

[14] Oschowski J, Bridwell KH, Lenke LG (2005). Neurological deficit from a purely vascular etiology after unilateral vessel ligation during anterior thoracolumbar fusion of the spine. Spine.

[15] Pappou IP, Papadopoulos EC, Swanson AN, Mermer MJ, Fantini GA, Urban MK (2006). Pott disease in the thoracolumbar spine with marked kyphosis and progressive paraplegia necessitating posterior vertebral column resection and anterior reconstruction with a cage. Spine.

[16] Kobayashi T, Kawahara N, Murakami H, Fujita F, Tomita K (2005). An experimental study on the influence of spinal shortening on the spinal cord. Jpn Orthop Assoc Congr Book.

[17] Toribatake Y (1993). The effect of total en bloc spondylectomy on spinal cord circulation. J Jpn Orthop Assoc.

